# Proteomic analysis implicates that postovulatory aging leads to aberrant gene expression, biosynthesis, RNA metabolism and cell cycle in mouse oocytes

**DOI:** 10.1186/s13048-022-01045-6

**Published:** 2022-10-14

**Authors:** Chuanxin Zhang, Xueqi Dong, Xinyi Yuan, Jinzhu Song, Jiawei Wang, Boyang Liu, Keliang Wu

**Affiliations:** Center for Reproductive Medicine, Shandong University, 250012 Jinan, Shandong China

**Keywords:** Postovulatory aging, Proteomics, Epigenome, Biosynthesis, RNA alternative splicing, Cell cycle

## Abstract

**Background:**

In mammals, oocytes display compromised quality after experiencing a process of postovulatory aging. However, the mechanisms underlying are not yet fully understood. Here, we portrayed a protein expression profile of fresh and aging metaphase II (MII) mouse oocytes by means of four-dimensional label-free quantification mass spectrometry (4D-LFQ).

**Results:**

The analysis of 4D-LFQ data illustrated that there were seventy-six differentially expressed proteins (DEPs) between two groups of MII stage oocytes. Fifty-three DEPs were up-regulated while twenty-three DEPs were down-regulated in the MII oocytes of the aging group, and Gene Ontology (GO) analysis revealed that these DEPs were mainly enriched in regulation of gene expression, biosynthesis, RNA metabolism and cell cycle. Our detailed analysis revealed that the expression of proteins that related to gene expression processes such as transcription, translation, post-translational modifications and epigenome was changed; the relative protein expression of RNA metabolic processes, such as RNA alternative splicing, RNA export from nucleus and negative regulation of transcription from RNA polymerase II promoter was also altered.

**Conclusion:**

In conclusion, we identified considerable DEPs and discussed how they agreed with previous researches illustrating altered protein expression associated with the quality of oocytes. Our research provided a new perspective on the mechanisms of postovulatory aging and established a theoretical support for practical methods to control and reverse postovulatory aging.

**Supplementary Information:**

The online version contains supplementary material available at 10.1186/s13048-022-01045-6.

## Background

It is well known that mammals oocytes are the foundation of reproductive biology. Mature oocytes stagnated at meiotic metaphase II (MII) are fertilized in an optimal time window after ovulation, namely 8–12h for mice and within 24h for humans [1]. Otherwise, it undergoes a time-dependent process of deterioration in the oviduct (in vivo) or culture media (in vitro) regarded as postovulatory aging [2, 3]. The degradation of oocyte quality due to postovulatory aging was associated with decreased fertilization rates, compromised embryo quality, implantation failure, early abortion and the birth of unhealthy offspring in various species [4–8].

A diverse variety of undesirable variations were demonstrated in the aging oocytes, including zona pellucida hardening, organellar dysfunction, spindle and chromosome abnormalities and aberrant regulation of molecular and biochemical events [6, 9]. Although in vivo and in vitro postovulatory aging resemble each other in manifestations, in vitro postovulatory aging leads to more extreme alterations on oocytes as the culture medium does not fully mimic the in vivo environment [6]. Accumulating evidences showed that oxidative stress [5, 10], altered Ca2 + homeostasis [1, 11], deadenylation of poly(A) tails of maternal effect genes [12] and epigenomic aberrations [13] were involved in the aging process. However, the precise molecular causes account for the series of changes in postovulatory aging oocytes remain to be further elucidated.

Since protein is the executor of vital biological processes, the variation of protein abundance plays an important role in the embryonic development [14]. A considerable series of correlative processes are demanded to maintain the abundance of cellular proteins, spanning the transcription, post-transcriptional processing and degradation of mRNAs to the translation, posttranslational modification and destruction of the proteins themselves [15–17]. Previous study by Jia, Bao-Yu et al. revealed that the function of endoplasmic reticulum and Golgi apparatus, two important organelles that play an important role in protein synthesis and processing, was significantly impacted in the aging oocytes. Thus, we supposed that the abundance of proteins in the aging oocytes may be broadly disturbed [2]. In order to comprehensively explore the molecular mechanisms of postovulatory aging, it has become an inevitable trend of current research to detect protein expression levels.

Mass spectrometry (MS) has become one of the major tools to detect and quantify a large quantity of proteins. The last few years have seen the birth of 4D-LFQ, a momentous breakthrough in proteomics technology. Apart from time, m/z and ion strength, the incorporation of ion mobility as an additional separation dimension into MS significantly improves the sensitivity of the measurement [18, 19]. Meanwhile, the application of parallel accumulation serial fragmentation (PASEF) greatly improves the scanning speed. The high sensitivity and speed of trapped ion mobility quadrupole time-of-flight mass spectrometer (timsTOF Pro) allow us to drastically increase measurement throughput and decrease sample amount, especially suitable for the detection of limited sample amounts and has been applied to tear fluid, plasma, sporozoite and so on. [19–21]. However, this technology has never been conducted on oocytes before. Considering that in vitro postovulatory aging can be easily influenced by the ingredients of culture media, in the current study, we performed the 4D-LFQ on in vivo postovulatory aging mouse oocytes model for the first time to map the dynamic proteomic changes between fresh and aging oocytes of mouse.

## Materials and methods

### Oocyte collection

Mice of Institute of Cancer Research (ICR) breed bought from Beijing Weitong Lihua Co., LTD were cultivated under standard conditions (12h dark and 12h light cycle, food and water ad libitum) in this experiment. To induce superovulation, 6- to 8- week-old female mice were injected intraperitoneally with 5 IU pregnant mare serum gonadotropin (PMSG) (110,914,564, Ningbo Sansheng Biological Technology Co., LTD), followed by 5 IU human chorionic gonadotropin (hCG) (110,911,282, Ningbo Sansheng Biological Technology Co., LTD) 48h later. The superovulated mice were humanly sacrificed at 13 and 25h after hCG injection, and the cumulus oocyte complexes (COCs) released from the oviductal ampullae were denuded of cumulus cells by pipetting with a thin pipette in a drop of M2 medium (M7167; Sigma-Aldrich) containing 0.1% hyaluronidase (H3506; Sigma-Aldrich) and collected as fresh and aging oocytes respectively.

### Protein extraction and trypsin digestion

The zona pellucida of oocytes was removed with acid Table’s solution (MR-004-D, Sigma-Aldrich) and washed with PBS (KGB5001, keyGEN bioTECH). The oocytes were transferred to a 1.5mL centrifuge tube, and 100 oocytes were collected as a group. The aging and fresh oocytes were both collected for three biological replicates, refrigerated at -80℃, shipped on dry ice.

Samples were taken out from − 80℃, a certain volume of lysis buffer consist of 8M urea (Sigma-Aldrich, USA) and 1% Protease Inhibitor Cocktail (Merck Millipore, Germany) was added, followed by sonication via ultrasound. The samples were centrifuged at 12,000×g at 4°C for 10min and the cell fragments were removed. The supernatant was transferred to a new centrifuge tube. An appropriate volume was taken and directly dyed with silver glue (Solarbio, China).

For digestion, the protein solution was reduced with 5mM dithiothreitol (Sigma-Aldrich, USA) for 30min at 56°C and alkylated with 11mM iodoacetamide (Sigma-Aldrich, USA) for 15min at room temperature in darkness. The sample was then diluted by adding 100mM TEAB (Sigma-Aldrich, USA) till urea concentration was less than 2M. Finally, trypsin (Promega, USA) was added at a 1:50 trypsin-toprotein mass ratio overnight and then a 1:100 trypsin-to-protein mass ratio for 4h.

### Liquid chromatography-mass spectrometry analysis and database search

The tryptic peptides were dissolved in 0.1% formic acid and 2% acetonitrile (solvent A) and separated by NanoElute ultra-high performance liquid system. The gradient consisted of an increase from 6 to 24% solvent B (0.1% formic acid in 100% acetonitrile) in 70min, 24–35% in 14min and climbing to 80% in 3min then holding at 80% for the last 3min, all at a constant flow rate of 450 nL/min. The peptides were injected into Capillary ion source for ionization at 2.0kV and analyzed by timsTOF Pro mass spectrometry. The peptide parent ions and their secondary fragments were detected and analyzed using high-resolution TOF. The m/z scan range was 100–1700 for full scan. After a first-order mass spectrometry collection, the secondary spectra with the charge number of parent ions in the range of 0–5 were collected in parallel cumulative serial fragmentation (PASEF) mode for 10 times with 30s dynamic exclusion.

The Secondary mass spectrometry data were retrieved using the Maxquant search engine (v.1.6.15.0) from the Mus_musculus_10090 database concatenated with the reverse decoy database. Trypsin/P was set as a cleavage enzyme and the number of missing cleavages was set as 2. The mass tolerance for precursor ions was set as 20 ppm in the first search and 20 ppm in the main search, and the mass tolerance for fragment ions was set as 20 ppm. Carbamidomethyl on Cys was set as a fixed modification and acetylation modification and oxidation on Met were set as variable modifications. The false discovery rate (FDR) was specified as 1%, and the minimum length for modified peptides was set as 7.

The expression values of each protein were calculated as the expected number of fragments per kilobase of transcript sequence per millions base pairs sequenced (FPKM). When p-value ≤ 0.05 and fold change > 1.5, the protein would be considered differentially expressed. Gene Ontology (GO, http://www.geneontology.org/) analysis, Kyoto Encyclopedia of Genes and Genomes (KEGG, http://www.kegg.jp/) analysis and eukaryote clusters of orthologous groups (KOG, http://www.ncbi.nlm.nih.gov/COG) classification of the enrichment of DEPs were performed. An adjusted P-value < 0.05 indicated the significant protein enrichment.

### Immunofluorescence microscopy

In general, at room temperature, oocytes were fixed with 4% paraformaldehyde in PBS for 30min, and permeabilized with 0.3% Triton-100 (T8787, Sigma-Aldrich) in PBS for 20min, then blocked with 1% BSA (0332, amresco) in PBS containing 0.1% Tween-20 (P9416, Sigma-Aldrich) and 0.01% Triton − 100 for 30min. Next, oocytes were incubated with primary antibodies in 1% BSA for 1h. After being washed three times in PBS containing 0.1% Tween-20 and 0.01% Triton − 100 with each time for 5min, oocytes were incubated with secondary antibody in PBS containing 0.1% Tween-20 and 0.01% Triton − 100 for 30min. Similarly, after being washed three times, oocytes were transferred onto slides in PBS containing 0.1% Tween-20 and 0.01% Triton − 100 and detected under a laser scanning confocal microscope (Dragonfly, Andor Technology, UK).

The primary antibodies were listed as follows: MTX2 (1:100 in dilution, 11610-1-AP, Proteintech), GDF9 (1:100 in dilution, ab93892, Abcam), TACC3 (1:50 in dilution, ab134154, Abcam), EIF5A (1:50 in dilution, 11309-1-AP, Proteintech), ASPM (1:150 in dilution, 26223-1-AP, Proteintech). The secondary antibodies were presented as follows: Goat Anti-Rabbit IgG H&L (Alexa Fluor 488) preadsorbed (1:500 in dilution, ab150081, Abcam), Goat Anti-Rabbit IgG H&L (Alexa Fluor 594) (1:500 in dilution, ab150080, Abcam), DAPI (1:500 dilution, D3571, Life Technologies).

### Statistical analysis

Images of oocytes labeled with the same antibody were captured under the identical scan setups. The mean fluorescence intensity in each oocyte was measured using ImageJ software (National Institutes of Health, Bethesda, MD, USA). Fluorescence values of fresh oocytes were arbitrarily set as 1, and values of aging oocytes were fixed relative to that of the fresh oocytes. The results were described as the mean ± SEM (standard error of mean). Statistical comparisons were performed using two-tailed Student’s t-test or Chi-square test. P-values < 0.05 were considered significant (*p < 0.05; **p < 0.01; ***p < 0.001).

## Results

### Global proteomics characteristics between fresh and aging mouse oocytes

**Fig. 1:**
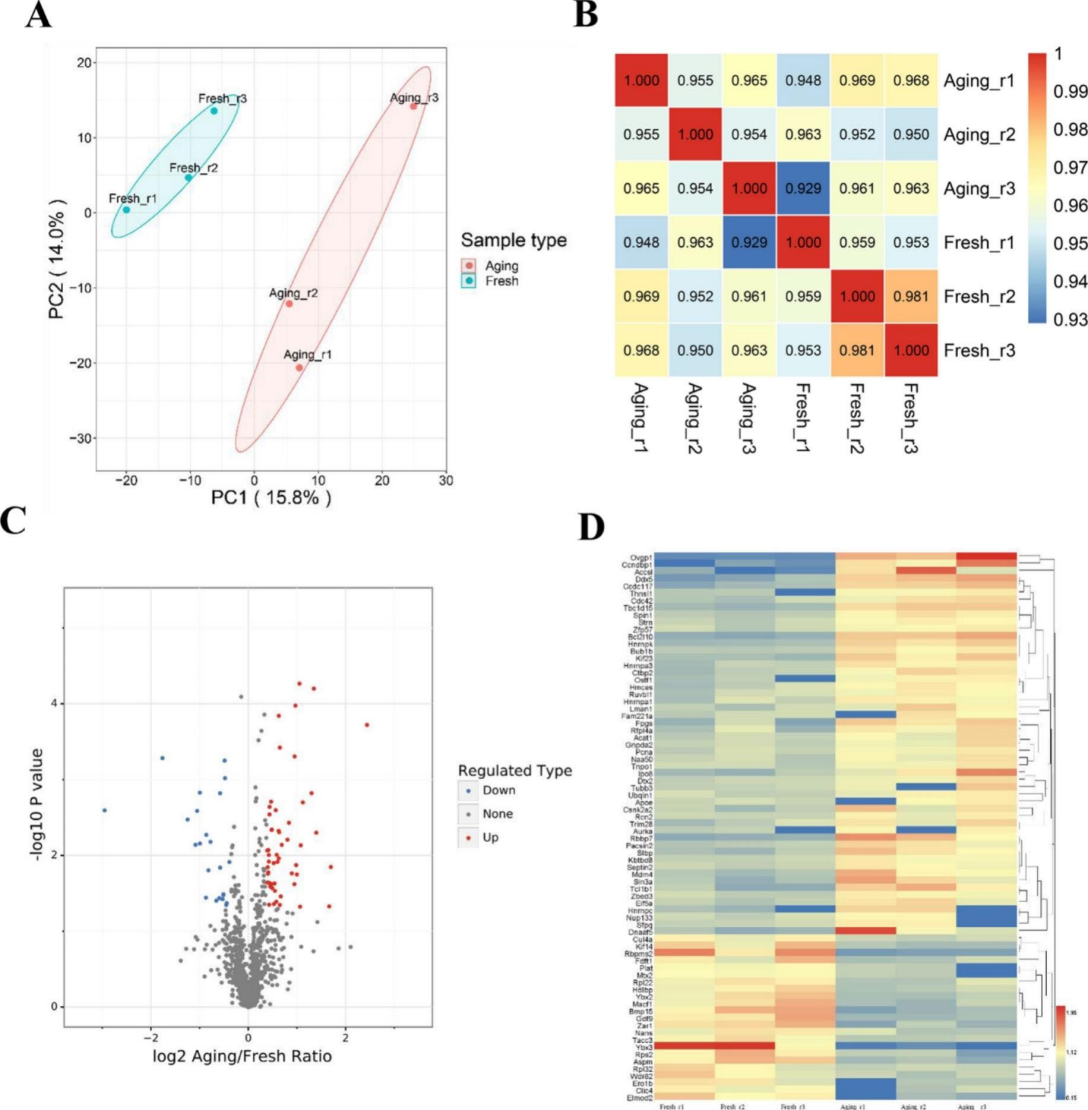
Protein expression levels of fresh and aging mouse MII oocytes. **a** Quantitative principal component analysis of all samples of fresh and aging oocytes. **b** Spearman’s correlation heatmap showing the consistency between repetitive samples and the discrepancy between groups. Color gradient indicates the magnitude of the correlation coefficient. **c** DEPs between oocytes of Fresh and Aging are shown in the volcano map. Proteins that express higher (up-regulated) in aging oocytes are shown in red, and proteins that are lower (down-regulated) are shown in blue. **d** Expression profiles heatmap of 76 DEPs in fresh and aging oocytes with a color gradient for gene abundance ranks

In this study, mouse oocytes collected 13h after hCG injection were regarded as fresh oocytes while those obtained 25h after hCG injection were seen as aging oocytes. To determine the differences in protein expression between fresh and aging oocytes, three biological replicates of each group were subjected to 4D-LFQ. First, principal component analysis was used to assess the protein intra-group repeatability. The results of biological and technical replicates were statistically consistent, and the aging oocytes were clearly distinguished from the fresh group (Fig. 1A). The spearman correlation coefficients between biological replicates were greater than 0.95, indicating that the biological repeat of mass spectrum (MS) exhibited highly reproducible results (Fig. 1B). A total of 11,438 peptides were identified by spectrogram analysis, among which 11,089 were specific peptides. According to this criterion, the total protein number identified was 1,887, of which 1,266 were quantifiable. The details of all identified proteins as well as the peptides were provided in **Table S1**. Subsequently, totally seventy-six differentially expressed proteins (DEPs) were screened out by log2 (Fold Change) greater than 1.3, among which 53 were up-regulated and 23 down-regulated (Fig. 1C and 1D). Therefore, we found that aging oocytes possessed a significantly different protein expression patterns compared with fresh oocytes.

**Fig. 2:**
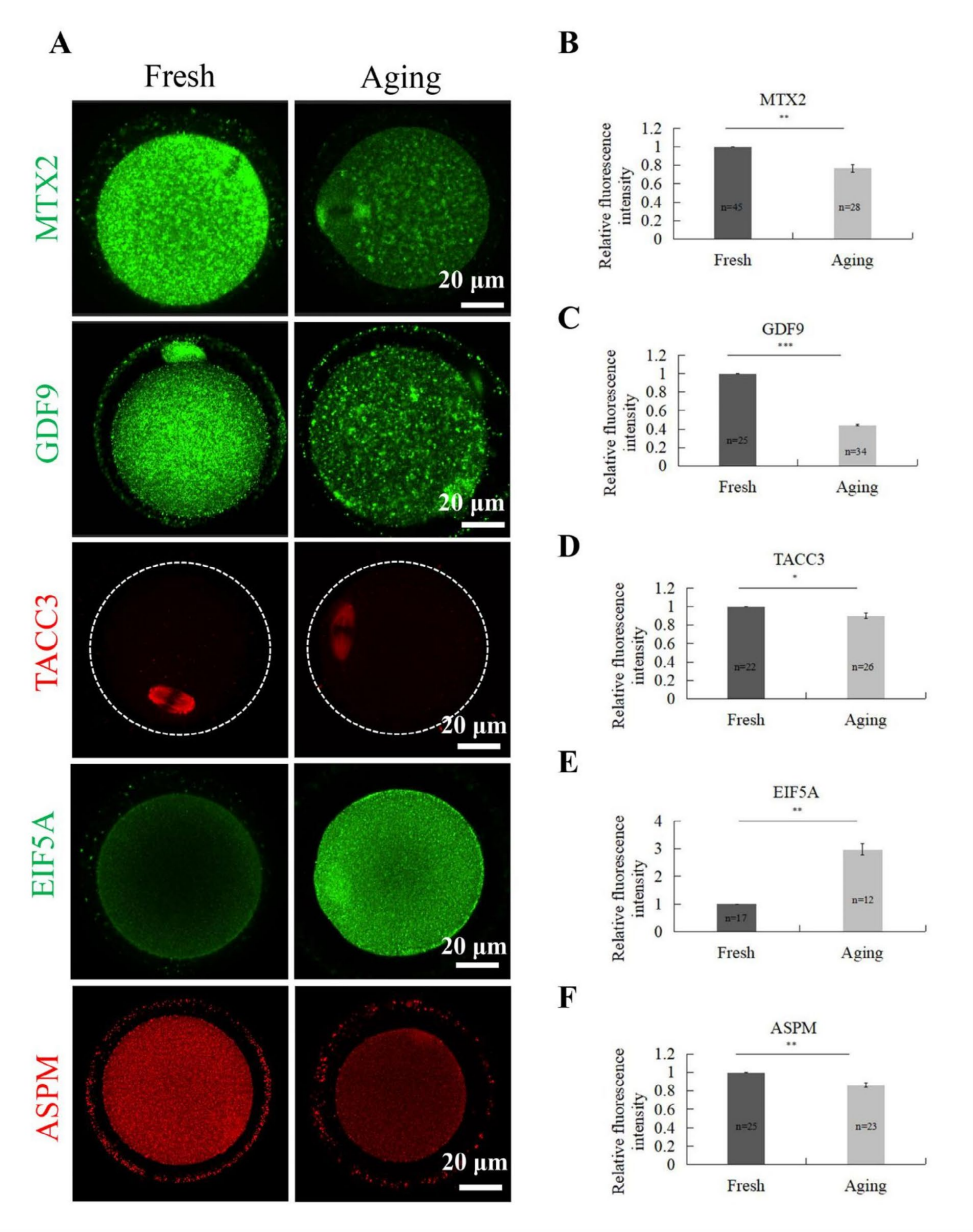
Validation of DEPs by immunofluorescence. **a** Representative confocal images of fluorescent staining of five candidate DEPs in fresh and aging oocytes, namely MTX2, GDF9, TACC3, EIF5A and ASPM. Scale bar, 20μm. **b** Intracellular relative fluorescence intensity of MTX2 signal intensity in fresh and aging oocytes. **c** Intracellular relative fluorescence intensity of GDF9 signal intensity in fresh and aging oocytes. **d** Intracellular relative fluorescence intensity of TACC3 signal intensity in fresh and aging oocytes. **e** Intracellular relative fluorescence intensity of EIF5A signal intensity in fresh and aging oocytes. **f** Intracellular relative fluorescence intensity of ASPM signal intensity in fresh and aging oocytes. *p<0.05, **p<0.01, ***p<0.001 indicate significant differences

To verify the proteomics results, samples of both fresh and aging oocytes were further analyzed by immunofluorescence. We chose metaxin-2 (MTX2), growth/differentiation factor 9 (GDF9), transforming acidic coiled-coil-containing protein 3 (TACC3), eukaryotic translation initiation factor 5A-1 (EIF5A) and abnormal spindle-like microcephaly-associated protein homolog (ASPM) as candidates in the experiment, and the results were consistent with the 4D-LFQ analysis (Fig. 2A-F).

### GO, KEGG and KOG analysis of DEPs for fresh and aging oocytes

**Fig. 3:**
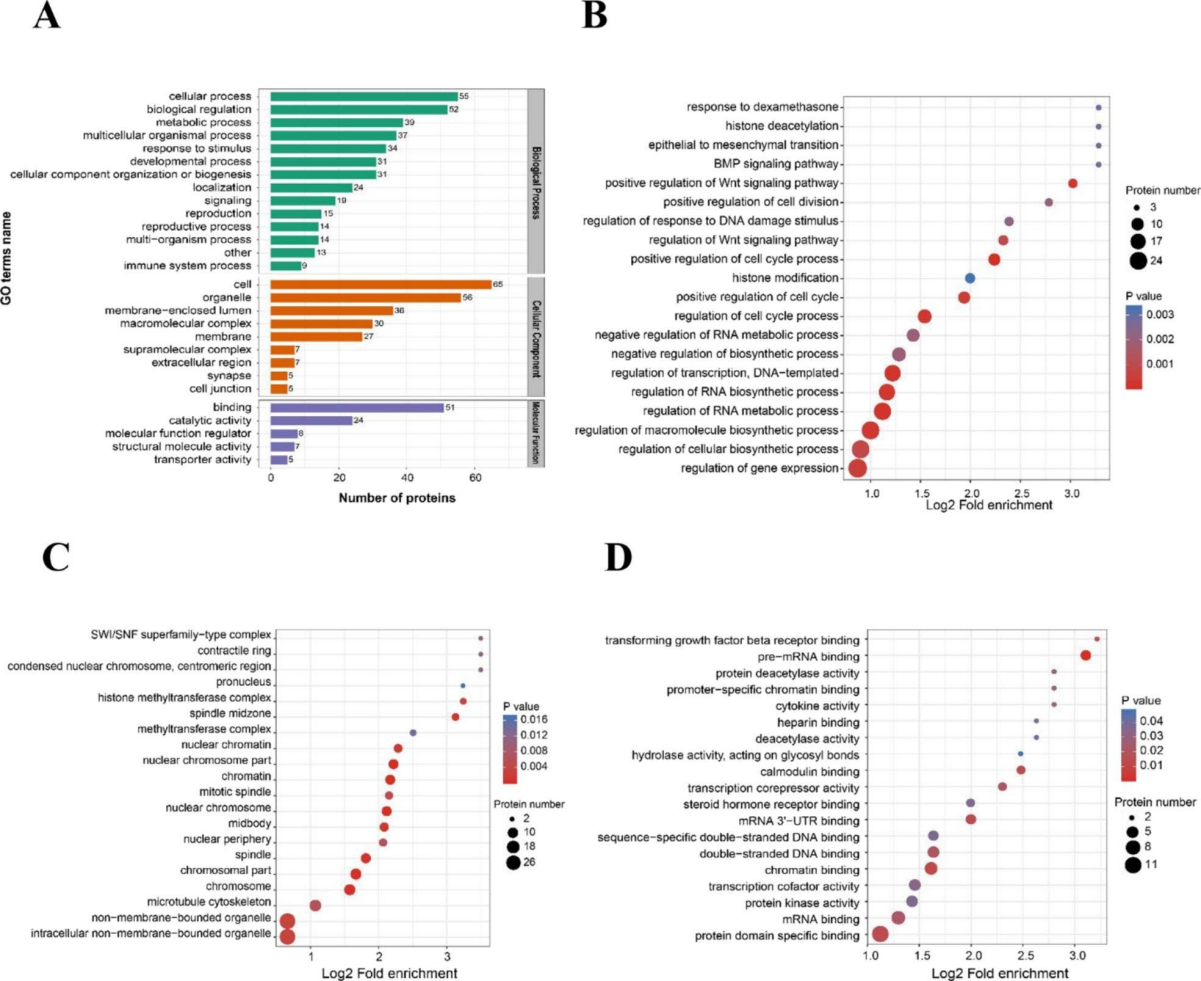
GO analysis of differentially expressed proteins (DEPs) between fresh and aging mouse MII oocytes. **a** GO (Gene Ontology) enrichment analysis on DEPs. Green refers to terms relating to biological processes, orange refers to terms relating to cellular components and purple refers to terms relating to molecular function. **b** The top 20 GO terms related to biological process were presented in the enrichment analyses of DEPs within the aging oocytes. **c** The top 20 GO terms related to cellular components were presented in the enrichment analyses of DEPs within the aging oocytes. **d** The top 20 GO terms related to molecular function were presented in the enrichment analyses of DEPs within the aging oocytes

To better understand the functions the DEPs performed during postovulatory aging, the DEPs were enriched into GO terminology. GO analysis showed that alterations of protein expression were related to manifold biological processes, cellular components and molecular functions (Fig. 3A). “Cellular process” and “biological regulation” were revealed with salient differences in biological processes category, including 56 and 52 DEPs, respectively. “Cell” and “organelle” were shown to be interfered in cellular components category, involving 65 and 56 DEPs, respectively. “Binding” and “catalytic activity” showed a significantly close relationship in molecular functions category, including 51 and 24 DEPs, respectively. In terms of biological processes, the most enriched GO terms fell into four major classes, regulation of gene expression, regulation of biosynthetic process, regulation of RNA metabolic process and regulation of cell cycle process, which were the main focuses in the subsequent parts (Fig. 3B). For cellular components, the two most enriched terms were intracellular non-membrane-bounded organelle and non-membrane-bounded organelle (Fig. 3C). And as to molecular functions, protein domain specific binding was the most enriched term (Fig. 3D).

**Fig. 4:**
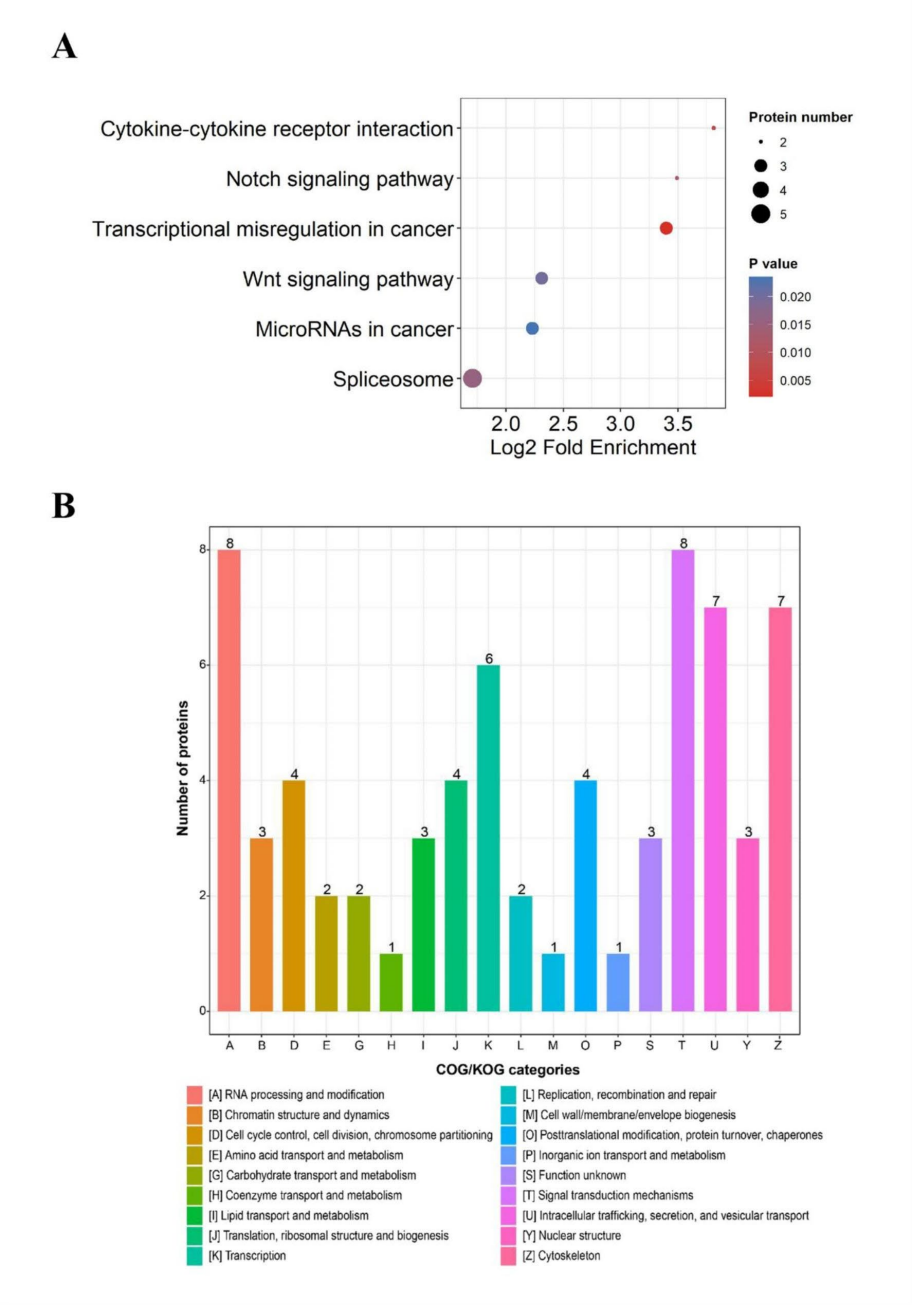
KEGG and KOG analysis of differentially expressed proteins (DEPs) between fresh and aging mouse MII oocytes. **a** The top KEGG pathways were presented in the enrichment analyses of DEPs within the aging oocytes. **b** Histogram representing clusters of orthologous groups (KOG) classification. The legend on the bottom showed a description of the 18 functional categories

The Kyoto Encyclopedia of Genes and Genomes (KEGG) classification system serves as an alternative functional annotation of proteins in accordance with their related biochemical pathways. Regardless of whether the protein was up-regulated or down-regulated, the most significant enrichment was the “spliceosome” pathway (Fig. 4A).

To further verify the effectiveness of the annotation process, we identified the unigene numbers with eukaryote clusters of orthologous groups (KOG) classification. Altogether, there were 69 unigenes identified to 18 categories (Fig. 4B). Among these categories, “RNA processing and modification” and “Signal transduction mechanism” accounted for the largest proportion (8, 11.6%) followed by “Intracellular trafficking, secretion, and vesicular transport” and “cytoskeleton” (7, 10.1%), and then “transcription” (6, 8.7%).

### Abnormal regulation of gene expression during the process of postovulatory aging

**Fig. 5:**
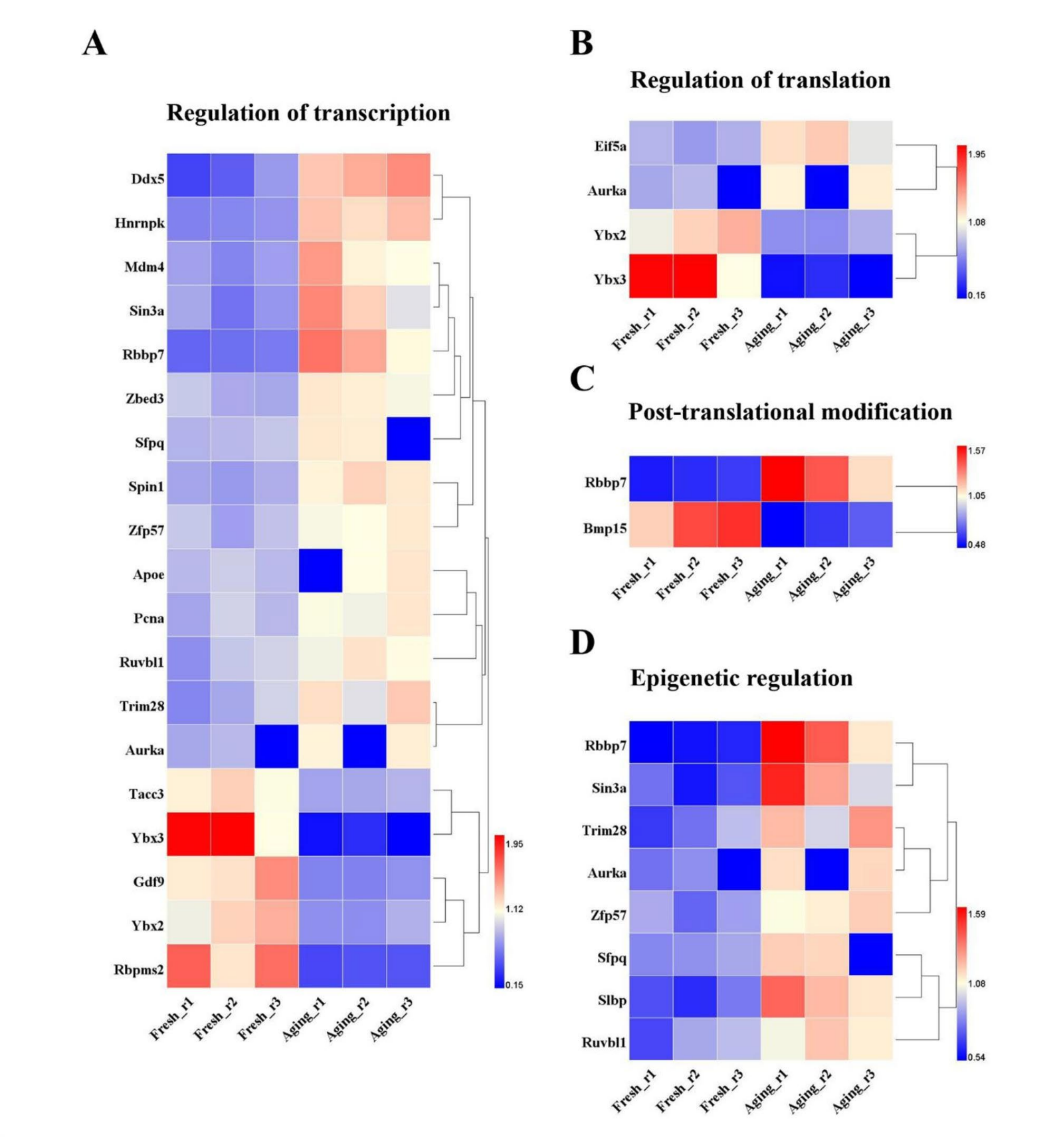
Abnormal regulation of gene expression during the process of postovulatory aging. **a** The heat map for the protein expression level related to the regulation of transcription. **b** The heat map for the protein expression level related to the regulation of translation. **c** The heat map for the protein expression level related to the regulation of post-translational modification. **d** The heat map for the protein expression level related to the epigenetic regulation

In order to function accurately and timely, oocyte strictly regulates its gene expression to meet the specific protein demands at given moments. Briefly, there are three major stages along the protein production pathway that an oocyte can perform gene regulation: transcription, translation and post-translational modifications respectively [22]. Among DEPs related to regulation of gene expression, twenty were annotated with regulation of “transcription” associated GO terms including DEAD box helicase 5 (DDX5), heterogeneous nuclear ribonucleoprotein K (Hnrnpk) and so on; four were annotated with “regulation of translation” GO term including Eif5a, aurora kinase A (Aurka), Y-box-binding protein 2 (Ybx2) and Y-box-binding protein 3 (Ybx3); and two were annotated with “post-translational protein modification” GO term including retinoblastoma binding protein 7, chromatin remodeling factor (RBBP7) and bone morphogenetic protein 15 (Bmp15), indicating all stages of gene regulation were influenced by postovulatory aging (Fig. 5A-C).

Meanwhile, establishment of the epigenetic asset also plays an essential role in the preservation of cell identity and the regulation of gene expression, which is indispensable for normal development after fertilization. [1, 23]. In our study, eight up-regulated DEPs were found to be related with epigenetic modification based on “histone modification”, “methylation” or epigenetic-associated GO terms, including RBBP7, transcriptional regulator, SIN3A (Sin3a), tripartite motif-containing 28 (Trim28), Aurka, zinc finger protein 57 (Zfp57), splicing factor proline/glutamine rich (Sfpq), stem-loop binding protein (SLBP) and RuvB-like 1 (Ruvbl1) (Fig. 5D).

### Interference in regulation of RNA metabolic process during the process of postovulatory aging

**Fig. 6:**
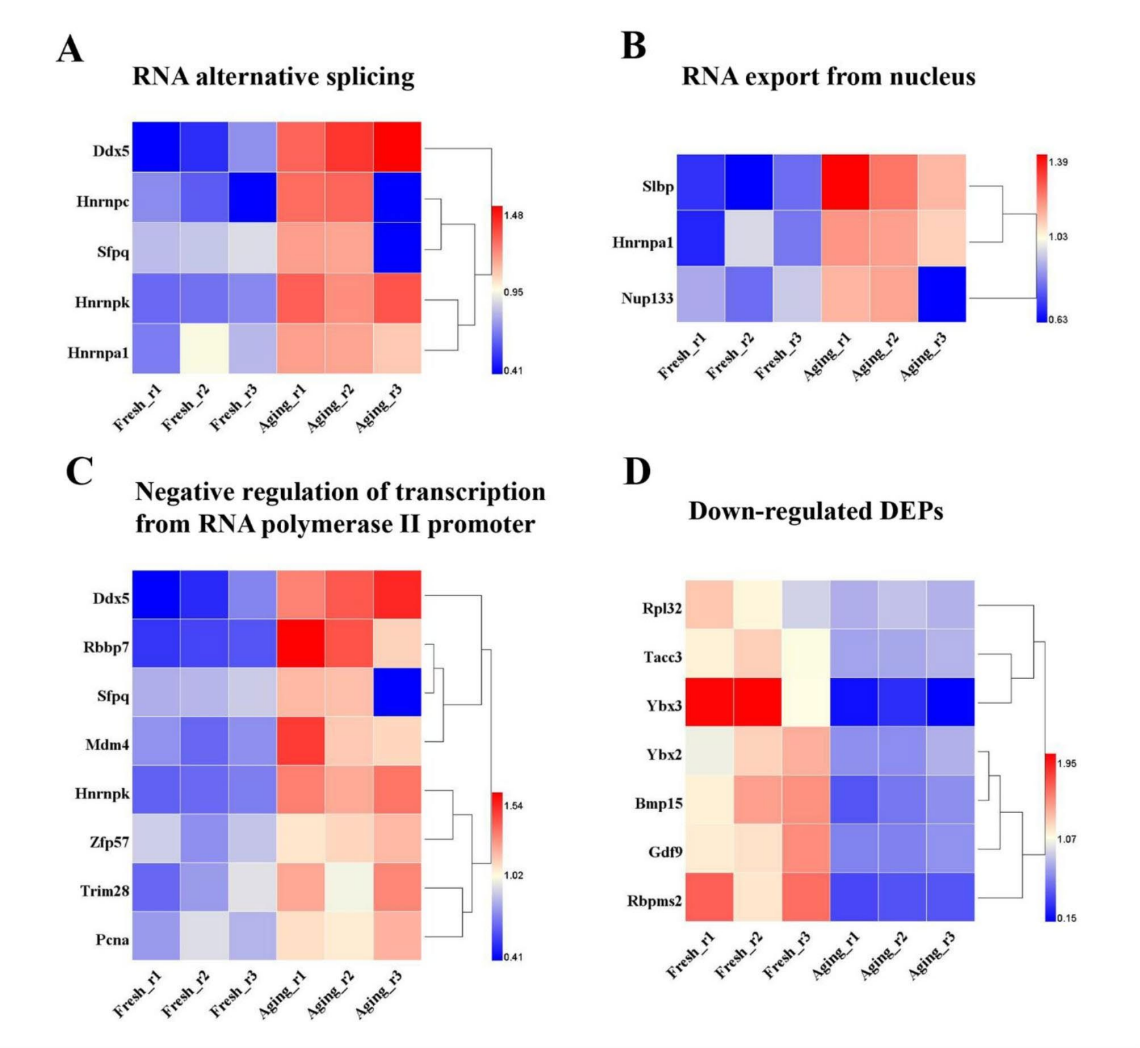
Interference in regulation of RNA metabolic process during the process of postovulatory aging **a** The heat map for the protein expression level related to RNA alternative splicing. **b** The heat map for the protein expression level related to RNA export from nucleus. **c** The heat map for the protein expression level related to negative regulation of transcription from RNA polymerase II promoter. **d** The heat map for the down-regulated protein expression level related to the regulation of RNA metabolic process

By information retrieval using the National Center for Biotechnology Information (NCBI) [24], we initially divided the up-regulated proteins related to regulation of RNA metabolic process into three categories: RNA alternative splicing, RNA export from nucleus and negative regulation of transcription from RNA polymerase II promoter, respectively (Fig. 6A-C). Among these proteins, five DEPs were splicing-related proteins based on “RNA splicing” or spliceosome-associated GO terms [25], including Ddx5, Hnrnpc, Hnrnpk, heterogeneous nuclear ribonucleoprotein A1 (Hnrnpa1) and Sfpq (Fig. 6A);three DEPs participated in RNA export from nucleus including Slbp, Hnrnpa1 and nucleoporin 133 (Nup133) (Fig. 6B); eight DEPs functioned in negative regulation of transcription from RNA polymerase II promoter, respectively Ddx5, Rbbp7, Hnrnpk, Sfpq, Trim28, Zfp57, proliferating cell nuclear antigen (Pcna) and transformed mouse 3T3 cell double minute 4 (Mdm4) (Fig. 6C).

The seven down-regulated proteins associated with “regulation of RNA metabolic process” GO term were presented in Fig. 6D. It is worth noting that the abundance of the germ-cell specific RNA-binding protein YBX2 (also known as MSY2), which plays a central role in ensuring the stability of mRNAs and their poly(A) tail [26], was significantly reduced.

Taken together, our analyses suggest that postovulatory aging has a differential influence on RNA splicing, RNA export from nucleus, transcription and RNA stability, which appears to one of the causations of declined quality and developmental potential in the aging oocytes.

### **Altered regulation of biosynthetic process and cell cycle process due to postovulatory aging**

**Fig. 7:**
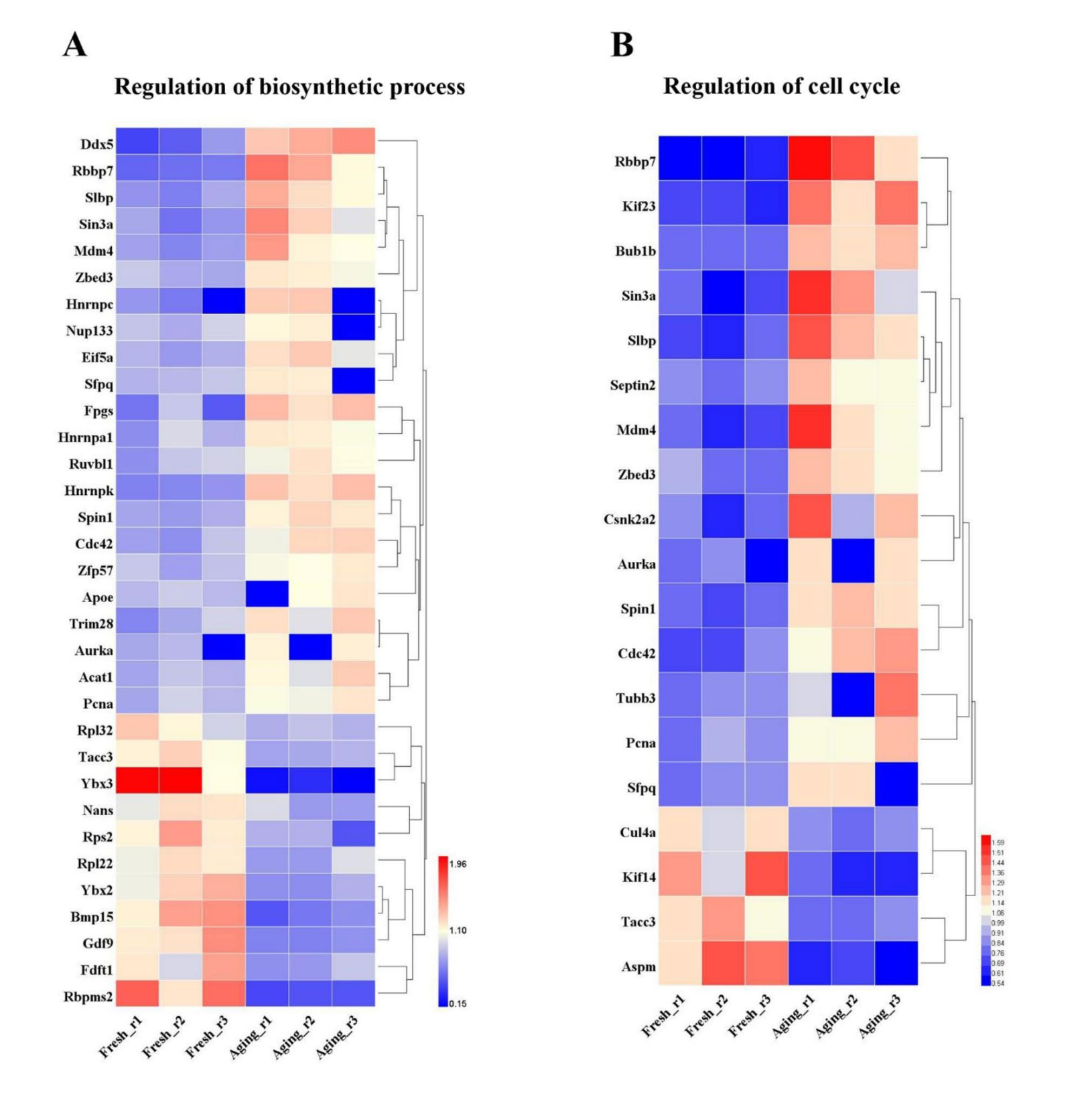
Altered regulation of biosynthetic process and cell cycle process due to postovulatory aging. **a** The heat map for the protein expression level related to the regulation of biosynthetic process. **b** The heat map for the protein expression level related to the regulation of cell cycle

Apart from an important maternal genetic contribution to each progeny, oocytes also provide a large library of macromolecules for offspring to maintain early embryogenesis and control necessary metabolic functions [27, 28]. Intriguingly, a total of 33 proteins differentially expressed were biosynthesis-related based on “regulation of biosynthetic process” associated GO terms, among which twenty-two showed an increased expression pattern, while eleven exhibited a reduced expression level (Fig. 7A). Among these DEPs, there were two pivotal oocyte-secreted factors, Gdf9 and Bmp15, both of which belong to the transforming growth factor-beta (TGFβ) superfamily. It has been reported that the combination of Gdf9 and Bmp15 acts as activator in the process of steroidogenesis [29]. The down regulation of the two proteins might interfere with the synthesis of steroid hormones and thus disrupt the endocrine and paracrine communications between cumulus cells and the oocyte.

Numerous cell cycle-related defects were observed in the aging oocytes including abnormal length of spindle, depolymerized astral microtubules, chromosomal misalignments, premature chromosome separation and so on [1, 9]. Likewise, we found that up to 19 proteins differentially expressed were involved in regulation of cell cycle. Fifteen of them were up-regulated, while only four showed a decreased expression pattern (Fig. 7B).

## Discussion

The declining quality of oocytes due to postovulatory aging is a major problem in reproductive medicine as fertilization of obtained mature oocytes can be postponed on account of various unforeseeable circumstances such as increased workload or delayed accessibility of semen samples [2, 6]. Therefore, significant importance should be attached to figure out the underlying mechanisms and potential methods to control and reverse the process. Taking it into account that protein is the ultimate executor of molecular functions, we conducted 4D-LFQ to investigate the protein expression profile alterations during mice postovulatory aging.

A total of 1266 quantitive proteins were identified in this study. Among the 76 proteins (6%) that were significantly differentially expressed, 53 (4.2%) showed up-regulation expression in the aging group while 23 (1.8%) were downregulated. Compared to previous study by Jiang et al. a decade ago, which reported 26 DEPs in vitro in aging porcine oocytes using two-dimensional Difference Gel Electrophoresis (2D DIGE) together with Matrix-Assisted Laser Desorption/Ionization Time of Flight/Time of Flight Mass Spectrometry (MALDITOF-TOF MS) [30], we identified significantly more DEPs with more advanced 4D-LFQ technology, let alone that more DEPs, varying with different culture media, should be found refer to in vitro postovulatory aging. To further understand the impact of postovulatory aging on oocytes, the DEPs were enriched into the GO, KEGG and KOG analysis, starting with biological processes, cellular components and molecular functions, pathways and unigenes respectively. The results of GO analysis showed that most DEPs were related to regulation of gene expression, biosynthetic process, RNA metabolic process and cell cycle process. Then we mainly focused on DEPs involved in these four biological processes.

Firstly, we filtrated the DEPs participated in regulation of gene expression. Conspicuously, proteins were found to be differentially expressed in regulation of each of the three major stages of gene expression, that is, transcription, translation and post-translational modifications, which suggested that postovulatory aging affected every stage of gene expression. In the last decades, researches have manifested that postovulatory aging can disrupt the normal epigenetic configuration before and after fertilization, thus interfering with embryo epigenome and its subsequent development [31, 32]. Histone modifications are among the most crucial epigenetic factors that regulate this process [6]. Evidence showed that augment of acetylation on histones H3 and H4 as well as hypomethylation of H19 could accelerate the aging process [31, 33]. In the current study, six proteins associated with histone modification exhibited a differential expression, confirming the epigenetic variations due to postovulatory aging at the global proteomic level. Excitingly, Sin3a and Ruvbl1, both associated with histone acetylation, were significantly up-regulated. Moreover, the overexpression of another maternal-effect factor SLBP, which acts as a stabilizer of histone mRNA, could lead to an overload of histone H3 on chromosomes, resulting in chromosome excessive condensation [1, 34]. Trim 28 and Zfp57, both belong to maternal-effect proteins and act synergistically on DNA methylation of early embryo during mouse oocyte to embryo transition [35, 36], showed an increasing expression. Our results provided insights into molecular mechanisms of epigenetic abnormalities caused by postovulatory aging.

It is generally acknowledged that mammalian early embryos are transcriptionally quiescent, the development depends on maternal RNAs stored in the oocyte cytoplasm until zygotic genome activation [14, 37]. Therefore, the metabolism of RNA is critical in the mature MII oocyte before fertilization. Our research showed that proteins related to RNA alternative splicing, RNA export from nucleus and negative regulation of transcription from RNA polymerase II promoter were significantly expressed. Based on the available evidences, alternative splicing plays a key role in regulating early embryonic development, especially the transition from zygote to 2-cell stages [38, 39]. Previous research also demonstrated that the hyperphosphorylated form (IIO) of RNA polymerase II, which takes charge of transcription of mRNA in eukaryotes, was found to be colocalized with mRNA splicing proteins and operate on mRNA splicing [40]. Given that critical processing including alternative splicing of exons takes place within the nucleus and then RNAs are exported to the cytoplasm to participate in further gene expression [41], the up-regulation of RNA export-related genes may have a causality with more frequent splicing. Thereout, we speculated that abnormal alternative splicing could be one of the important reasons accounting for the decline in embryonic developmental potential of aging oocytes [42].

The healthy development of early embryos relies on the synthesis of numerous macromolecules. Our data identified 33 DEPs involved in the regulation of biosynthesis, including the biosynthetic process of coenzyme, amino acid, cofactor, nucleobase-containing compound and so on, which are all necessary and indispensable factors in the progression of embryonic development. Hence, we suspected that the disturbance in the regulation of biosynthetic process might result in the impaired developmental competency of aging oocytes.

Cell cycle regulation is considered to be one of the known mechanisms of postovulatory aging, and the downregulation of maturation-promoting factor (MPF) and mitogen-activated protein kinase (MAPK) proteins and mitotic arrest deficient 2 (Mad2) transcript were strongly linked to the process [43, 44]. Yet the specific biochemical pathway has not been clearly elaborated. Thrilly, up to 19 DEPs identified played a part in the regulation of cell cycle, and some of these proteins are pivotal molecular assuring the proper progression of the cell cycle in oocytes. Spindlin 1 (Spin1), component of a ribonucleoprotein complex and regulates meiotic resumption in mouse oocytes [45], was overexpressed in the aging oocyte. A critical spindle checkpoint protein budding uninhibited by benzimidazole 1 (Bub1) that ensures accurate chromosome alignment and homolog separation in oocyte meiosis [46, 47] was also up-regulated in the aging oocytes. Similarly, the key acentriolar microtubule-organizing center (MTOC) constituent Aurka which plays a vital part in regulating meiotic spindle assembly in oocytes [48–50] also exhibited an increased expression pattern. The expression levels of several microtubule-associated proteins including TACC3, KIF14 and ASPM were also reduced in the aging oocytes. The results of our study will provide direction for the future in-depth study of this mechanism of postovulatory aging.

Among the DEPs screened out, five were identified oocyte-specific maternal-effect factors, namely T cell leukemia/lymphoma 1B, 1 (Tcl1b1), Slbp, Trim28, Zfp57 and zygote arrest 1 (Zar1), which affected embryonic development via different pathways and perhaps acted as a culprit in the compromised embryonic development of aging oocytes as well [36].

The limitation of our study is that we did not perform overexpression or knockdown verification of these DEPs on fresh mouse matured MII oocytes. In addition, although postovulatory aging is a relatively conserved progress among species, there are differences between the proteome of mice and humans. We used mouse oocytes to explore processes related with postovulatory aging due to the rarity of human oocyte samples, which may not absolutely mirror processes in aging human oocytes. Well-designed verification researches are demanded to further test our hypotheses.

## Conclusion

In conclusion, with the application of 4D-LFQ, this study revealed that abnormal regulation of gene expression, RNA metabolism, biosynthesis and cell cycle might all accelerate the course of postovulatory aging and lead to the decline in the developmental potential of aging mouse oocytes. These findings in mouse oocytes may, in part, explain disturbances and declined developmental potential of aging human oocytes and help to prevent postovulatory aging in vivo and in vitro. A deeper insight into these DEPs is expected to open up a new angle on the mechanisms of postovulatory aging, providing a theoretical basis for developing viable strategies to improve natural pregnancy and ART success rates.

## Electronic supplementary material

Below is the link to the electronic supplementary material.

Supplementary Material 1: Table S1. Detail information of all MS identified proteins and peptides.

## Data Availability

MS data will be uploaded to pubmed if this manuscript was accepted. All other data that support the findings of this study are available from the corresponding authors upon reasonable request.

## Abbreviations

2D DIGE: two-dimensional Difference Gel Electrophoresis
4D-LFQ: four-dimensional label-free quantification mass spectrometry
ASPM: abnormal spindle-like microcephaly-associated protein homolog
Aurka: aurora kinase A
Bmp15: bone morphogenetic protein 15
Bub1: benzimidazole 1
COCs: cumulus oocyte complexes
DDX5: DEAD box helicase 5
DEP: differentially expressed protein
EIF5A: eukaryotic translation initiation factor 5A-1
FDR: false discovery rate
FPKM: fragments per kilobase of transcript sequence per millions base pairs sequenced
GDF9: growth/differentiation factor 9
GO: Gene Ontology
hCG: human chorionic gonadotropin
Hnrnpa1: heterogeneous nuclear ribonucleoprotein A1
Hnrnpk: heterogeneous nuclear ribonucleoprotein K
ICR: Institute of Cancer Research
KEGG: Kyoto Encyclopedia of Genes and Genomes
KOG: eukaryote clusters of orthologous groups
Mad2: mitotic arrest deficient 2
MALDITOF-TOF MS: Matrix-Assisted Laser Desorption/Ionization Time of Flight/Time of Flight Mass Spectrometry
MAPK: mitogen-activated protein kinase
Mdm4: transformed mouse 3T3 cell double minute 4
MII: metaphase II
MPF: maturation-promoting factor
MS: mass spectrum
MTOC: acentriolar microtubule-organizing center
MTX2: metaxin-2
NCBI: National Center for Biotechnology Information
Nup133: nucleoporin 133
PASEF: parallel accumulation serial fragmentation
Pcna: proliferating cell nuclear antigen
PMSG: pregnant mare serum gonadotropin
RBBP7: retinoblastoma binding protein 7, chromatin remodeling factor
Ruvbl1: RuvB-like 1
SEM: standard error of mean
Sfpq: splicing factor proline/glutamine rich
Sin3a: transcriptional regulator, SIN3A
SLBP: stem-loop binding protein
Spin1: Spindlin 1
TACC3: transforming acidic coiled-coil-containing protein 3
Tcl1b1: T cell leukemia/lymphoma 1B, 1
TGFβ: transforming growth factor-beta
timsTOF Pro: time-of-flight mass spectrometer
Trim28: tripartite motif-containing 28
Ybx2: Y-box-binding protein 2
Ybx3: Y-box-binding protein 3
Zar1: zygote arrest 1
Zfp57: zinc finger protein 57
